# The interaction between future food considerations, regulation of eating behaviors, sense of purpose, and social media consciousness among college students: a study on mediation effects and network analysis

**DOI:** 10.3389/fpsyg.2026.1827219

**Published:** 2026-08-12

**Authors:** Lu Wang, Jiayu He, Xiaoming Zhang, Heqing Wang, Jingmin Song, Ningbo Chen, Xinhong Zhu

**Affiliations:** 1 School of Nursing, Hubei University of Chinese Medicine, Wuhan, China; 2 Hubei Shizhen Laboratory, Wuhan, China

**Keywords:** consideration of future, eating behaviors, network analysis, sense of purpose, social media

## Abstract

**Background:**

The purpose of this study was to investigate the association between future food consideration and regulation of eating behaviors among college students, using chain mediation and network analysis (NA), with a specific focus on employing chain mediation and NA to examine the associations linking sense of purpose and social media consciousness.

**Methods:**

A cross-sectional study was conducted among college students (*n* = 778) with questionnaires, including Regulation of Eating Behaviors Scale (REBS), the Consideration of Future Consequence-food (CFC), the Appearance-Related Social Media Consciousness Scale (ASMC) and the Sense of Purpose Scale with Adults (SOP). Statistical analyses included mediation modeling and NA.

**Results:**

Three levels of eating behaviors motivation were identified, including high level of motivation (29.82%), medium level (58.10%), low level (12.08%). Compared to high level of motivation, low level of motivation was correlated with a lower likelihood of the CFC (OR: 0.853, 95% CI: 0.791 to 0.920), SOP (OR: 0.882, 95%CI: 0.851 to 0.914), ASMC (OR: 0.966, 95%CI: 0.944 to 0.988). Furthermore, SOP (effect: 0.102, 95%CI: 0.070 to 0.136) and ASMC (effect: −0.027, 95%CI: −0.048 to −0.007) were associated with CFC and partially accounted for variance in REBS in the total population. However, in the terms of normal weight population, the association involving ASMC (effect: −0.016, 95%CI: −0.044 to 0.009) was not significant. NA results indicated that A18, C6 and A19 were centrally positioned within the network. In addition, B5 and B4 were considered bridging symptoms in the NA.

**Conclusion:**

CFC was associated with REBS among Chinese college students, with SOP and ASMC partially explained the observed association. This study also identifies A18, C6, and A19 as central nodes within the network. In addition, B5, B4, and B1 are recognized as bridge symptoms connecting CFC, SOP, ASMC, and REBS. Furthermore, central symptoms (A18, C6, and A19) and bridge symptoms (B5 and B4) may serve as potential targets for future interventions.

## Introduction

Dietary motivation is strongly linked to health, yet eating behaviors are shaped by multifaceted factors (Musaiger et al., 2017; Naeem et al., 2024). Only 7% of students reported leading a very active lifestyle, and 4% exhibited fairly good knowledge of nutrition (Yahia et al., 2015). Poor diets can lead to mental health problems (Schweren et al., 2021) and chronic diseases (Kianersi et al., 2023; Wu et al., 2023). Dietary pattern modification is now recognized as a pivotal strategy for sustainably nourishing the growing global population (Parodi et al., 2018). Although healthy eating is important for health, there is a certain degree of cognitive and behavioral dissonance in people’s healthy eating behaviors (Pelletier et al., 2016; Rodrigues et al., 2019). Individual motivation plays a key role in shaping food choices within social contexts, with health serving as a primary driver of dietary behavior. Evidence showed that young and obese people in urban areas show more pronounced emotional motivation and often make conscious choices of foods that may have adverse health effects (Ilić et al., 2023). College students who employ avoidance coping strategies may be particularly vulnerable to the impact of stress on emotional eating (Dalton, 2023). Adopting a healthier diet may be directly associated with a reduction in the severity of symptoms associated with insomnia (Yao et al., 2024). Despite growing interest in the intrinsic drivers of young adults’ dietary choices, the joint mediating roles of sense of purpose and social media consciousness remain underexplored, particularly among Chinese college students. This study uses a cross-sectional design to incorporate these constructs into an integrated framework, examining how personal meaning and digital awareness are associated with dietary behaviors in this cultural context.

Self-determination Theory (SDT) concentrates on the extent to which an individual’s behaviors is autonomous and self-directed (Mushtaq et al., 2023; Wilksch et al., 2019). It proposes a framework, consisting of six sub-theories, for understanding social contextual factors, self-regulation and integration, and motivation to engage in a variety of behaviors, including within the context of health (Ng et al., 2012). Within SDT, behavioral motivation ranges from autonomous to controlled. Autonomous regulation reflects greater volition, whereas controlled regulation is driven by external or internalized pressure. SDT posits that individuals possess an inherent potential for psychological growth, which fosters self-actualization and optimal functioning. Notably, eating motivation was associated with a sense of purpose (Berkowitz et al., 2023), future considerations (Hill et al., 2017), and external social media (Rojo et al., 2023).

Consideration of Future Consequences (CFC) is a cognitive construct reflecting the extent to which individuals weigh immediate and distant consequences of current behaviors (Murphy et al., 2019; Petrocelli, 2003; Strathman et al., 1994). More specifically, a person thinks about the trade-off between near-term desires and far future benefits of a potential behavior when deciding whether or not to engage in a behavior (Rappange et al., 2009), which is used to predict a person’s behavior (Vilar et al., 2020) and examine prospective awareness (Schwarz, 2007). It reflects the extent to which individuals consider and are influenced by the potential long-term outcomes when making behavioral decisions (Pfund et al., 2024). Although future orientation research initially centered on adolescents, it is critical for adult health behaviors (Liu, 2020). Healthy lifestyles in college predict academic and health success (Özenoğlu et al., 2024). Similarly, a study using data from the Hawaii Study of Personality and Health suggested that a purpose-driven life may also be a healthier life (Hill et al., 2017). Focusing on the awareness of the future consequences may lead to healthier eating behaviors and construct eating behaviors (Bénard et al., 2024). Therefore, we hypothesize as follows: (Hypothesis 1) CFC will be positively related to REBS.

Sense of purpose (SOP) refers to the sense of thinking oriented to reaching goals for people who do things and make decisions, and is a meaningful goal and direction that individuals believe can guide their lives (Mohammad Johari and Tan, 2024). Within SDT, SOP is conceptualized as a quintessential form of autonomous regulation, reflecting behaviors driven by volition and intrinsic values. Purposive adults would tend to adopt more healthy behaviors such as engaging in activities and adopting positive and healthy eating habits (Hill et al., 2017). Nurturing a strong sense of meaning and future direction is positively associated with a stronger SOP, future meaning and direction. Such future orientation is positively associated with both SOP and self-development (Joshanloo, 2024). For example, it is recommended that individuals prioritize the cultivation of effective relationships and the management of stress in order to enhance one’s overall sense of wellbeing in the future (Kato et al., 2022). However, the associations between CFC and these outcomes remain underexplored. Hence, we hypothesize that: (Hypothesis 2) CFC and SOP will be positively related.

The phenomenon of appearance-related social media consciousness (ASMC), defined as a pervasive and persistent awareness of one’s physical attractiveness in online images, is prevalent among young adults and adolescents (Choukas-Bradley et al., 2020; Maheux et al., 2022). A study has indicated a correlation between the pursuit of an ideal athletic body type and frequent exposure to fitness content on online platforms (Flauzino et al., 2024). This phenomenon has been attributed to an escalating societal bias concerning body weight (Flauzino et al., 2024). Most young adults obtain health information from social media, both actively and through passive exposure. And they judged the credibility of sources by appearance and instinct (Lim et al., 2022). When the motivation of Internet users is rooted in their desire for a particular message, they may encounter content that is characterized by positive, courage and success (Jenkins et al., 2020). ASMC reflects the intersection of digital environments and body image concerns in today’s social media-saturated world, thus we hypothesize that SOP and ASMC will be positively related (Hypothesis 3).

The diversity and influence of social media have had an undeniable impact on individuals’ perceptions of body image and eating disorders. Roughly three-quarters reported frequent ASMC experiences among college women in the United States (Choukas-Bradley et al., 2019). According to SDT, behavioral motivation ranges from autonomous to controlled regulation. While autonomous regulation reflects greater volition, controlled regulation is driven by external or internalized pressure to conform to external standards. The recent research indicates that heightened ASMC functions as a controlled regulation, serving as a correlate of health issues (Choukas-Bradley et al., 2020), such as high level of C-reactive protein (CRP; Lee et al., 2022), body dissatisfaction and disordered eating behaviors (Yang et al., 2022). Eating disorders and over-attachment to social media represent a significant challenge for young people (Mushtaq et al., 2023; Wilksch et al., 2019), and there is a notable correlation between social media and eating disorders (Barakat et al., 2023). In addition, their combined effects can lead to elevated stress levels (Suhag and Rauniyar, 2024). Thus, we hypothesized that ASMC and REBS will be negatively related (Hypothesis 4).

**Figure 1:**
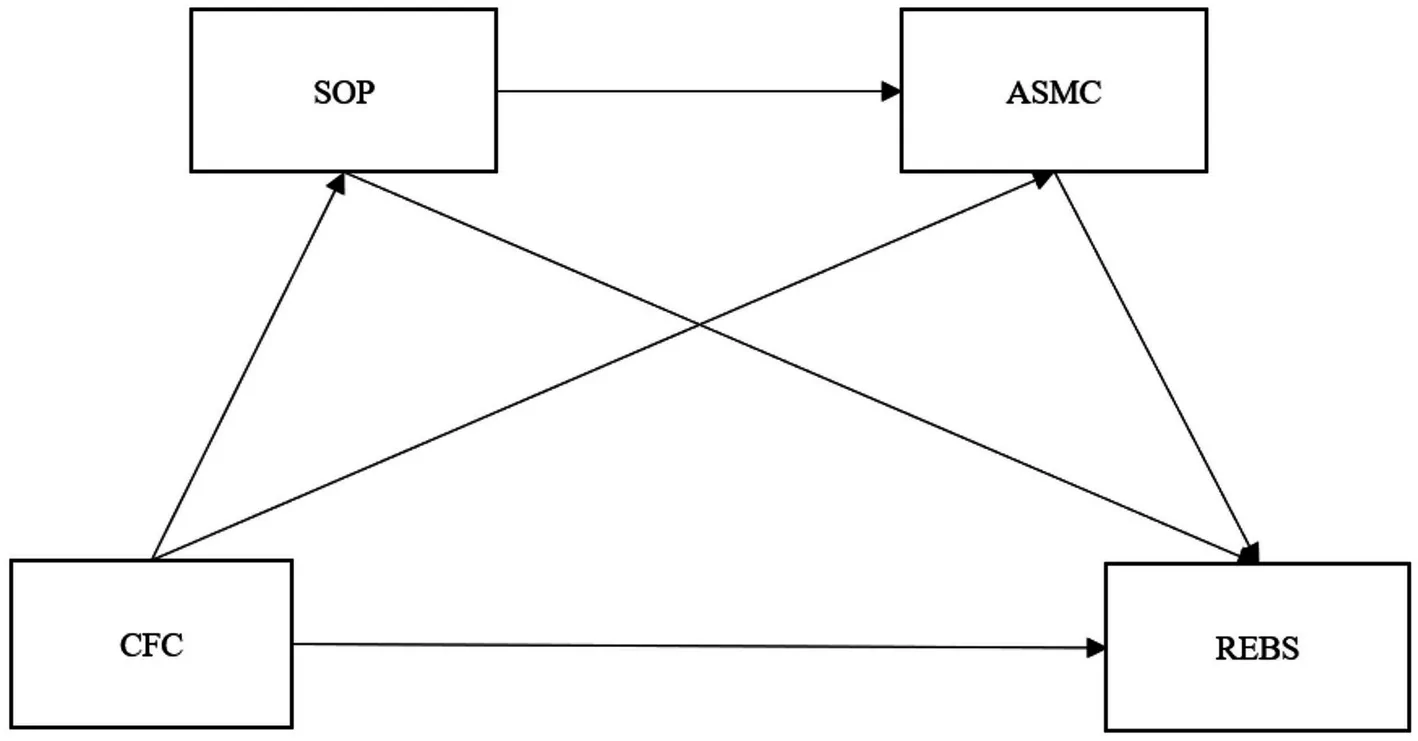
Hypothesized models of association involving CFC, SOP, ASMC, and REBS.

For college students, the relationship between CFC and REBS may involve various factors. Network analysis (NA) offers a comprehensive framework for exploring such complex associations, allowing for the systematic modeling of interrelationships among CFC, SOP, ASMC, and REBS. Therefore, this study proposes integrating mediation analysis with NA to enable a detailed examination of the associations among CFC to REBS. Specifically, the research intends to examine how SOP and ASMC partially explain the observed association between CFC and REBS (Figure 1). However, no study has explicitly situated these constructs within SDT or examined whether CFC is associated with REBS through its relationships with SOP and ASMC. By integrating SDT, the present study offers a theoretically grounded account of why considering future consequences is associated with autonomous forms of motivation and REBS. This theoretical linkage addresses an important gap in understanding how internal and external factors interact to underlie eating motivation. This approach aims to provide a theoretical basis for promoting health behaviors among college students.

## Methods

### Study design and population

A cross-sectional study was conducted among college students aged 18–28 years in Wuhan, Hubei Province, China from April to June 2024. Data were collected using an anonymous self-administered questionnaire developed and administered through the Wen Juanxing application, an online survey platform. The selection of specific demographic and lifestyle variables was guided by theoretical considerations and prior publications (Marentes-Castillo et al., 2024; Meng et al., 2025; You, 2024). The 70-item questionnaire comprised multiple-choice questions accounting for 95.71% and open-ended questions representing 4.29%. Participants were recruited via invitations distributed through university-affiliated WeChat groups and quick response (QR) codes. And this approach represented a convenience sampling method with voluntary participation. To enhance response rates, the platform employed a forced-response design requiring completion of all items. Prior to participation, all respondents provided electronic informed consent through checkbox confirmation after reviewing study details including research objectives, voluntary participation terms and confidentiality protocols.

The inclusion criteria were the (1) aged ≥18 years, (2) enrolled as a full-time or part-time student with valid institutional registration, (3) those who gave informed consent and participated in this study voluntarily. The exclusion criteria were the (1) respondents who are off college due to illness or leave of absence as well as mental illnesses, (2) significant functional cognitive impairment, (3) implausible completion times less than 120 s, (4) logical inconsistencies in responses (e.g., contradictory answers to validation questions). The study collected 820 questionnaires. During data preprocessing, invalid responses were excluded based on insufficient response time. Subsequently, patterned answering (e.g., “S-shaped” or “Z-shaped” trends) and extreme outliers were detected and removed. All items were then numerically coded and reverse-scored items were appropriately transformed. Following these rigorous procedures, 778 valid questionnaires were retained, yielding an effective response rate of 94.88%.

### Assessment tools

#### Questionnaires of demographic characteristics

The questionnaire items include age, gender, grade, body mass index (BMI), residence, monthly cost of living, smoking, drinking, physical activity, sedentary time, sleep duration and daily use of mobile phones. And the study adopts the BMI Criteria of weight for the Chinese (Criteria of Weight for Adults, 2013-04-18).

#### The regulation of eating behaviors scale

Luc Pelletier et al. (2004a, 2004b) developed the questionnaire, which translated by Di (Lihua, 2021), comprising five dimensions: internal motivation, integrative regulation, identity regulation, introjected regulation and external regulation. It is a 20-item scale used to assess current situation of dietary motivation, with each item containing seven response options (1 = Strongly Disagree, 7 = Strongly Agree). The total scores range from 20 to 140, with higher total scores indicating higher levels of motivation in individuals. In the present study, the internal consistency of REBS was quite acceptable (Cronbach’s *α* = 0.878) and 0.851 for the Chinese version.

#### The consideration of future consequence-food

Jannette et al. (van Beek et al., 2013) developed the questionnaire utilized for this study that was translated by Jiang (Hongyan, 2023), which consists of two dimensions: consideration of the present and the future, contains a total of 10 questions on a 5-point Likert scale that ranged from 1 = Strongly Disagree to 5 = Strongly Agree. The total scores range from 10 to 50, with higher total scores indicating higher levels of future consequence. In this study, the reverse dimension was 0.825 and the forward dimension was 0.804. Additionally, the Chinese version demonstrated strong internal consistency across different samples: in Sample 1, the reliability coefficients for the present and future dimensions were 0.86 and 0.82, respectively, while in Sample 2, they were 0.87 and 0.76.

#### The appearance-related social media consciousness scale

Choukas-Bradley et al. (2020) developed the questionnaire which was translated by Li (2023). The scale consists of 13 items. All responses are recorded on a 7-point Likert type scale from 1 = Never to 7 = Always. The total scores range from 13 to 91, with higher total scores indicating greater degree of the appearance-related social media consciousness. It was used to assess a reflection of the extent to which individuals are concerned about their ability to gain traction in social media audience groups and behaviors based on this, found to have good internal consistency reliability. In this study, the Cronbach *α* of the scale is 0.945. The Chinese version of the Cronbach’s α were 0.94 and 0.95 for males and females, respectively.

#### The sense of purpose scale with adults

Sharma et al. (2017) developed the questionnaire which was translated by Zhang (Wanying, 2022), and used to assess the degree of orientation, engagement, socialization, and motivation toward life goals and which consists of 14 items, was rated on a 7 statements from 1 = Never to 7 = Always. The total scores range from 14 to 98. Responses are summed, and higher scores indicating greater sense of purpose with adults. The Cronbach’ α for this study is 0.954, which is slightly higher than the total Cronbach’s α of the Chinese version of the scale 0.92.

## Data analysis

For continuous variables, normality was tested using the Shapiro–Wilk test. And depending on the type and distribution of the variable, the normally distributed was expressed as mean and standard deviation (SD). Group differences were assessed using one-way analysis of variance (ANOVA). To account for multiple comparisons, Levene’s test was first performed to evaluate the homogeneity of variances. If variances were equal (*p* > 0.05), the Least Significant Difference (LSD) test was applied. If variances were unequal (*p* ≤ 0.05), Welch’s ANOVA was used to determine the overall significance, followed by Tamhane’s T2 test for post-hoc pairwise comparisons. Categorical variables were summarized as frequencies and percentages. Group differences were evaluated using the Pearson Chi-square test when the total sample size was ≥40 and all expected cell counts were ≥5. Since only an overall association was examined, no Bonferroni adjustment or multiple comparisons were performed.

Latent class analysis (LCA) identified latent classes of participants based on total REBS scores. Estimation was conducted with the robust maximum-likelihood and expectation–maximization algorithms. Statistical fit indexes were used to assess model fit and to decide the final number of latent classes. The model that fit the data best was selected by a combination of the following criteria: (a) the Log-likelihood (LL), (b) the Akaike information criterion (AIC), (c) the Bayesian information criterion (BIC), (d) the Sample-Size Adjusted Bayesian Information Criteria (SSA-BIC), (e) the Lo–Mendell–Rubin Likelihood Ratio Test (LRT), (f) the Bootstrapped Likelihood Ratio Test (BLRT), and (g) entropy to be 0.6 or greater. However, rather than relying solely on the lowest values of these indices, we prioritized models that yielded theoretically meaningful, interpretable, and clinically plausible class solutions. Special attention was given to the size, distinctiveness, and substantive interpretability of each latent class. The final number of classes was therefore selected based on a combination of statistical evidence and conceptual coherence. Next, we conducted an unconditional multivariable logistic regression to identify sociodemographic and risk factors that predicted class membership.

Pearson’s correlation analysis was used to explore the correlation between the REBS, CFC, ASMC and SOP. Furthermore, logistic regression and linear regression were used to assess the relationship of REBS, CFC, ASMC and SOP. To ensure model stability, multicollinearity among covariates was assessed using variance inflation factors (VIF). Covariates included in the multivariable logistic regression model were those with a *p* < 0.05 in the ANOVA. Model 1: crude analysis without adjusting for any confounders. Model 2: adjusted for age and BMI. High level of motivation was chosen as the reference group and calculated the adjusted odds ratio (OR) for the primary endpoints were calculated for the other groups relative to this reference.

To evaluate the proposed mediational pathways, a structural equation model (SEM) was constructed to test the chain mediation hypothesis, incorporating CFC, REBS, SOP, and ASMC as key interrelated constructs. Model fit was assessed using the comparative fit index (CFI), Tucker–Lewis index (TLI), root mean square error of approximation (RMSEA), and the standardized root mean square residual (SRMR) (Hu and Bentler, 1999). The adjusted indices for this model included effect, bootstrap standard error (BootSE), low limit for confidence interval (LLCI) and upper limit for confidence interval (ULCI).

Subsequently, the network relationships among CFC, SOP, ASMC, and REBS were modeled using the Extended Bayesian Information Criterion Graphical Lasso (EBICglasso) algorithm in the R qgraph package (Hevey, 2018). In the R qgraph package, nodes represent variables and edges represent associations. Edges are positive (green) or negative (red), and the strength of the association between nodes is indicated by the thickness of the edge (Zhang et al., 2023). Centrality indices were measured using four metrics: strength, betweenness, closeness, and expected influence (EI). They were calculated to examine the relative importance and interconnectedness of variables within the network. To identify node centrality, this study employed EI due to its enhanced capability in networks with both positive and negative edges. This method offers a distinct advantage over the traditional strength centrality approach, which is predominantly based on edge weights (Bringmann et al., 2019). The stability of edge weights and centrality indices in the network was assessed using the “bootnet” package. Specifically, the stability of edge weights was reflected by the width of the 95% confidence intervals (CIs). CIs were calculated by a nonparametric bootstrap method, with narrower intervals indicating higher stability. The stability of centrality was assessed via the correlation stability coefficient (CS-C), interpreted as follows: a coefficient exceeding 0.25 indicates low stability, above 0.5 indicate adequate stability, and above 0.7 indicate high stability (Ye et al., 2024).

Data analyses were conducted using SPSS version 27.0, the PROCESS macro (version 4.2), and R version 4.5.0. LCA was performed in Mplus version 8.3, with bootstrapping set to 5,000 resamples. Network analysis was conducted in R using the qgraph package to estimate regularized partial correlation networks via the EBICglasso algorithm, with correlation matrices computed using the psych package. Network accuracy and stability were assessed using the bootnet package. Visualization of network structures was implemented via ggplot2. Mediation effects were evaluated based on whether the 95% confidence intervals (CIs) excluded zero, with CIs estimated via bootstrapping using 5,000 resamples in PROCESS. All tests were two-tailed and had a significance level of 0.05.

## Results

### Latent classes analysis of REBS

**Figure 2:**
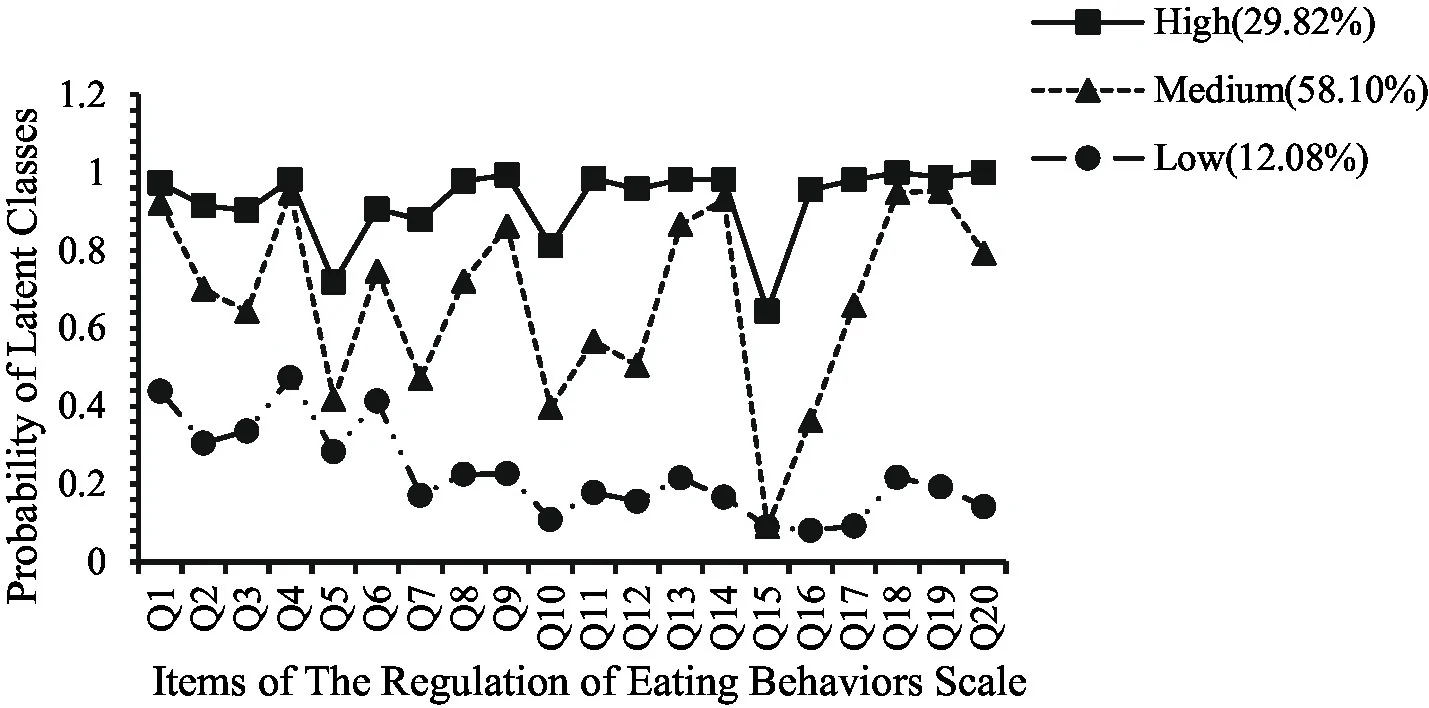
Conditional response probabilities for the 3-class models at each item of The Regulation of Eating Behaviors Scale (*N* = 778).

Table 1 shows the latent class models with 1–6 classes were calculated. We selected the three-class model as optimal based on a combination of fit indices and interpretability. While AIC, BIC, and SSA-BIC continued to decrease from 3 to 4 classes, the marginal gains in fit were offset by increasing model complexity. Critically, the LMR-LRT showed a significant improvement for the 3-class model over the 2-class model (*p* < 0.001). The entropy value remained high (0.897), suggesting clear classification accuracy. Furthermore, the 3-class solution yielded substantively meaningful and balanced class proportions without extremely small or redundant groups, balancing statistical adequacy with theoretical relevance. Three levels of motivation were identified (Figure 2). Cluster 1 (29.82%) labeled “High level of motivation,” showed the highest probability to take healthy behaviors. Participants in Cluster 2 (58.10%), labeled “Medium level of motivation,” showed moderate probabilities of adopting healthy eating behaviors. Cluster 3 (12.08%) had the lowest probability as compared to cluster 1 and 2 to take healthy measures, so we name it as “Low level of motivation.”

**Table 1:** The fitting statistics of latent class analysis of REBS (*N* = 778).

Number of classes	LL	AIC	BIC	SSA-BIC	Entropy	LMR-LRT	BLRT	Class probability (%)
1	−8,546.424	17,132.847	17,225.982	17,162.472				
2	−7,407.159	14,896.319	15,087.245	14,957.050	0.908	0.0000	0.0000	76.61/23.39
3	−7,011.601	14,147.202	14,435.919	14,239.038	0.897	0.0000	0.0000	29.82/58.10/12.08
4	−6,835.034	13,836.068	14,222.576	13,959.011	0.871	0.0000	0.0000	29.43/19.67/44.09/6.81
5	−6,732.755	13,673.510	14,157.809	13,827.559	0.843	0.1,151	0.0000	30.21/31.11/18.12/6.56/14.01
6	−6,657.608	13,565.215	14,147.306	13,750.370	0.852	0.0206	0.0000	29.95/29.05/20.31/11.31/5.91/3.47

LCA, latent class analysis; LL, log-likelihood; AIC, Akaike information criteria; BIC, Bayesian information criteria; SSA-BIC, sample-size adjusted Bayesian information criteria: LRT, the Lo–Mendell–Rubin likelihood ratio test; BLRT, the bootstrapped likelihood ratio test.

### General demographic characteristics of the study participants

**Figure 3:**
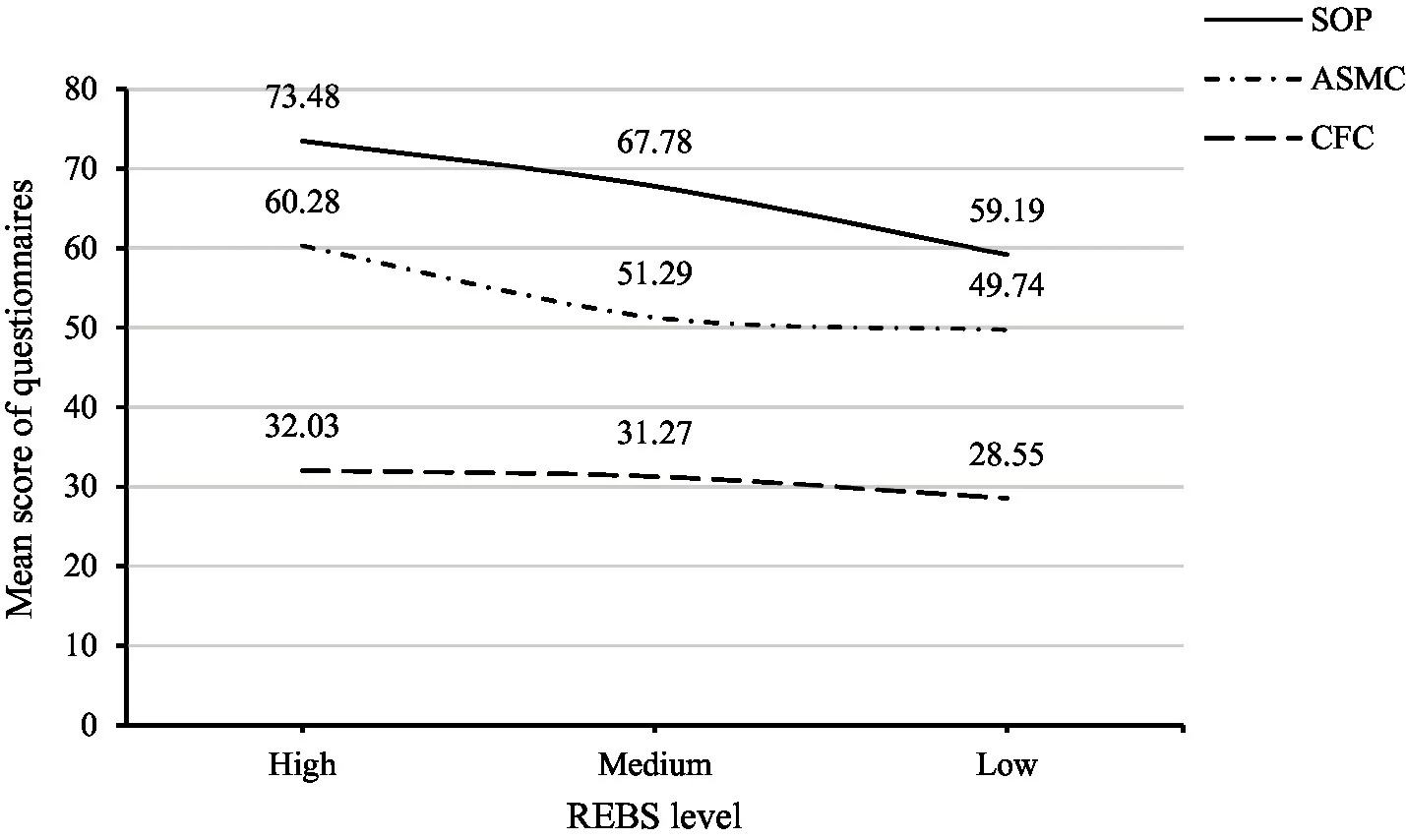
The mean scores of SOP, ASMC, and CFC across REBS (*N* = 778).

As Table 2 showed, the mean age of participants was (20 ± 2) years old, females accounted for 81.1% of the total, and the average BMI was (23.22 ± 6.82) with 13.5% of the population categorized as obese. Figure 3 showed the mean scores of SOP, ASMC and CFC across REBS. High REBS had the highest scores in SOP (73.48 ± 11.79), ASMC (60.28 ± 15.39) and CFC (32.03 ± 4.06). There were significant differences in gender, BMI, CFC, ASMC and SOP according to the REBS subgroups. For age, Welch’s ANOVA was not significant (*p* = 0.078) so the post-hoc tests were not performed. Significant group differences were observed in BMI, CFC, ASMC, and SOP. Low BMI was higher than medium/high BMI; low CFC was lower than medium/high CFC; high ASMC was higher than medium/low ASMC; and SOP increased stepwise (high > medium > low), with all pairwise comparisons statistically significant (Supplementary Table S1).

**Table 2:** Baseline characteristics (*N* = 778).

Variables	REBS			*F/χ^2^*	*p-*value
	High	Medium	Low		
Age, years, mean ± SD	20.31 ± 2.46	20.05 ± 1.41	20.11 ± 1.51	2.737	0.065
Gender, *n* (%)				8.938	0.011
Male	52 (22.4)	70 (15.5)	25 (26.6)		
Female	180 (77.6)	382 (84.5)	69 (73.4)		
Grade, *n* (%)				9.251	0.160
Freshman year	52 (22.4)	115 (25.4)	19 (20.2)		
Sophomore year	87 (37.5)	150 (33.19)	46 (48.9)		
Junior year	66 (28.50)	135 (29.87)	19 (20.2)		
Senior and above	27 (11.6)	52 (11.5)	10 (10.6)		
BMI	22.87 ± 6.22	22.90 ± 6.50	25.58 ± 9.01	6.537	0.002
Residence, *n* (%)				4.667	0.097
Rural	83 (35.8)	159 (35.2)	44 (46.8)		
Urban	149 (64.2)	293 (64.8)	50 (53.2)		
Monthly cost of living, *n* (%)				3.251	0.777
≤1,000 yuan	12 (5.2)	29 (6.4)	8 (8.5)		
1,000yuan~	49 (21.1)	107 (23.7)	20 (21.3)		
1,500yuan~	123 (53.0)	235 (52.0)	52 (55.3)		
≥2,000yuan	48 (20.7)	81 (17.9)	14 (14.9)		
Smoking, *n* (%)				5.421	0.247
Never	216 (93.1)	434 (96.0)	86 (91.5)		
Current smoking	13 (5.6)	13 (2.9)	5 (5.3)		
Smoking in the past	3 (1.3)	5 (1.1)	3 (3.2)		
Drinking, *n* (%)				1.039	0.904
Never	187(80.6)	365(80.8)	79(84.0)		
Current drinking	32 (13.8)	57 (12.6)	10 (10.6)		
Drinking in the past	13 (5.6)	30 (6.6)	5 (5.3)		
Physical activity, *n* (%)				1.641	0.440
Sufficient	131 (56.5)	235 (52.0)	47 (50.0)		
Insufficient	101 (43.5)	217 (48.0)	47 (50.0)		
Sedentary time, *n* (%)				1.116	0.572
<9 h	100 (43.1)	207 (45.8)	38 (40.4)		
≥9 h	132 (56.9)	245 (54.2)	56 (59.6)		
Sleep duration, *n* (%)				9.978	0.126
<6 h	13 (5.6)	26 (5.8)	9 (9.6)		
6–8 h	171 (73.7)	350 (77.4)	63 (67.0)		
8.1–9 h	43 (18.5)	64 (14.2)	15 (16.0)		
>9 h	5 (2.2)	12 (2.7)	7 (7.5)		
Daily use of mobile phones, *n* (%)				13.813	0.087
<2 h	5 (2.2)	5 (1.1)	4 (4.3)		
2–4 h	43 (18.5)	70 (15.5)	14 (14.9)		
4.1–6 h	80 (34.5)	157 (34.7)	31 (33.0)		
6.1–8 h	59 (25.4)	130 (28.8)	16 (17.0)		
>8 h	45 (19.4)	90 (19.9)	29 (30.9)		
CFC, mean ± SD	32.03 ± 4.06	31.27 ± 4.81	28.55 ± 3.60	20.602	<0.001
ASMC, mean ± SD	60.28 ± 15.39	51.29 ± 14.06	49.74 ± 11.37	35.135	<0.001
SOP, mean ± SD	73.48 ± 11.79	67.78 ± 10.90	59.19 ± 9.29	58.436	<0.001

### Association between REBS, CFC, ASMC and SOP

The correlation analysis of the influencing factors is presented in Supplementary Table S2. REBS was positively correlated with CFC, ASMC and SOP (*p* < 0.01) respectively. Compared to high level of REBS, participants in the medium motivation class was significantly associated with ASMC (OR: 0.947, 95%CI: 0.923 to 0.972) and SOP (OR: 0.956, 95%CI: 0.927 to 0.986; Table 3). REBS in the low level of motivation group had lower odds of ASMC (OR: 0.927, 95%CI: 0.886 to 0.970) and SOP (OR: 0.840, 95%CI: 0.789 to 0.894). All VIF values were below 5, indicating no significant multicollinearity (Supplementary Table S3). Meanwhile, a positive linear relationship was observed between the four variables (Supplementary Table S4).

**Table 3:** Multiple logistic regression analysis of factors related to REBS among college students (*N* = 778).

REBS level	Model 1^a^		Model 2^b^	
	OR	95%CI	OR	95%CI
Medium				
CFC	0.963	0.927–1.001	1.030	(0.963–1.102)
ASMC	0.961	(0.949–0.973)***	0.947	(0.923–0.972)***
SOP	0.972	(0.957–0.987)***	0.956	(0.927–0.986)**
Low				
CFC	0.856	(0.801–0.914)***	0.893	(0.777–1.026)
ASMC	0.962	(0.942–0.982)***	0.927	(0.886–0.970)**
SOP	0.888	(0.860–0.917)***	0.840	(0.789–0.894)***

^a^: crude model. ^b^: adjusted gender and BMI. *p < 0.05; **p < 0.01; ***p < 0.001.

### Chain mediated effects test

**Figure 4:**
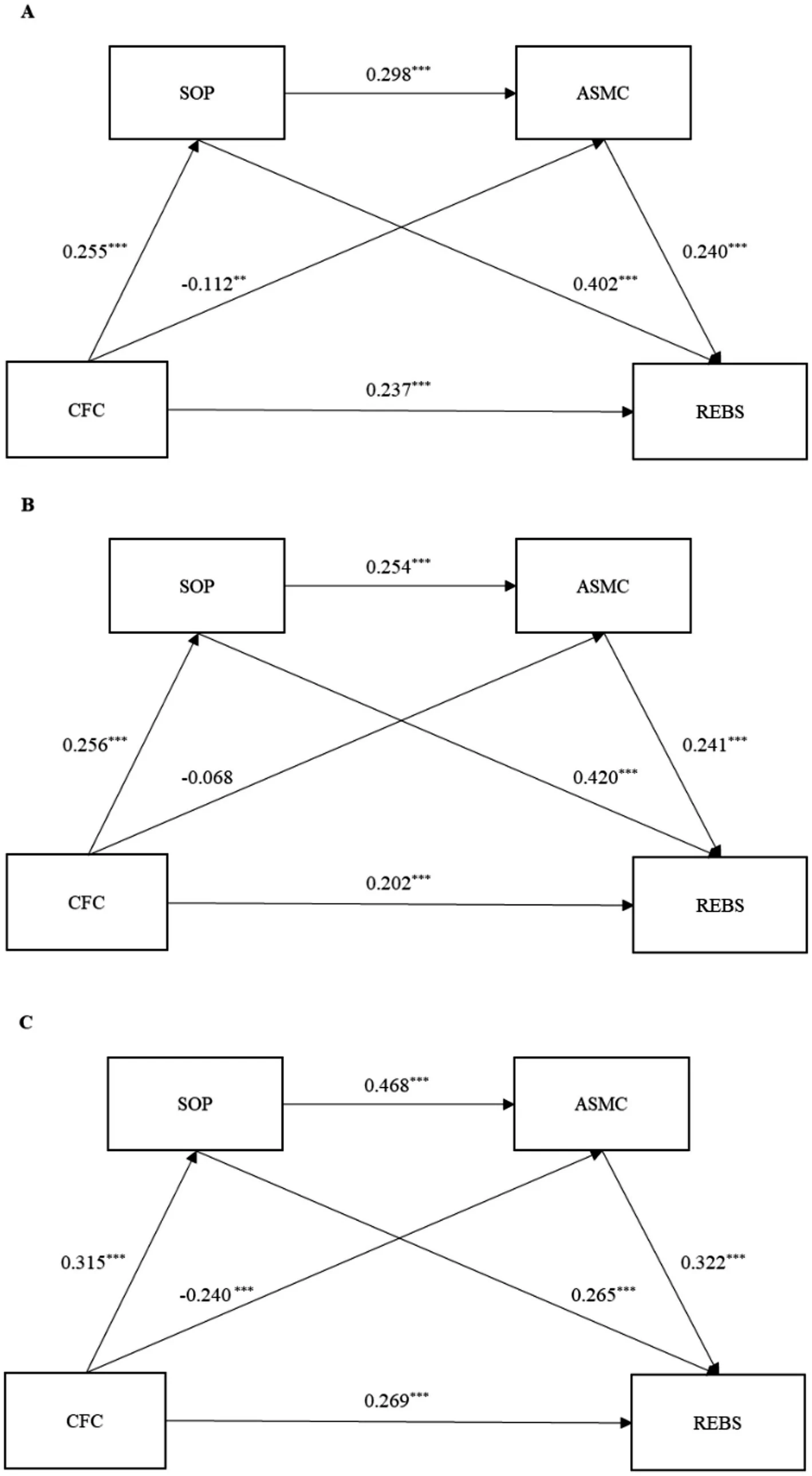
Chain Mediation Model of CFC and REBS. **(A)** Chain mediation model of the total participants (*N* = 778); **(B)** Chain mediation model of the normal weight participants (*N* = 422); **(C)** Chain mediation model of the overweight and obese participants (*N* = 202). **p < 0.01; ***p < 0. 001.

The mediation path model is shown in Figure 4. SOP and ASMC partially accounted for the variance in the association between CFC and REBS. As shown in Table 4, incorporating SOP and ASMC into the model revealed a significant total association between CFC and REBS (*β*: 1.051, 95% CI: 0.839 to 1.262). These constructs showed significant association: SOP (*β*: 0.102, 95% CI: 0.070 to 0.136) and ASMC (*β*: −0.027, 95% CI: −0.048 to −0.007). In the overweight and obese subgroup, the total association between CFC and REBS remained significant (*β*: 1.133, 95% CI: 0.670 to 1.597). Both SOP (*β*: 0.084, 95% CI: 0.030 to 0.160) and ASMC (*β*: −0.077, 95% CI: −0.133 to −0.026) showed significant indirect associations in this subgroup. The total sample model fit indices did not indicate a perfect fit (*χ*^2^/df = 4.43, CFI = 0.718, TLI = 0.707, RMSEA = 0.066, SRMR = 0.084), but they provided a tolerable approximation of the data structure sufficient (Supplementary Table S5).

**Table 4:** The effect of CFC on REBS mediated by SOP and ASMC (*N* = 778).

Group (number)	Path type	Path	Effect	BootSE	LLCI	ULCI
Total (*n* = 778)	Indirect effect	CFC → SOP → REBS	0.102	0.017	0.070	0.136
		CFC → ASMC→REBS	−0.027	0.011	−0.048	−0.007
		CFC → SOP → ASMC→REBS	0.018	0.005	0.010	0.028
	Total effect	CFC → REBS	1.051	0.108	0.839	1.262
Normal weight (*n* = 422)	Indirect effect	CFC → SOP → REBS	0.108	0.022	0.066	0.151
		CFC → ASMC→REBS	−0.016	0.013	−0.044	0.009
		CFC → SOP → ASMC→REBS	0.016	0.006	0.006	0.028
	Total effect	CFC → REBS	0.968	0.145	0.682	1.254
Overweight and obesity (*n* = 202)	Indirect effect	CFC → SOP → REBS	0.084	0.033	0.030	0.160
		CFC → ASMC→REBS	−0.077	0.028	−0.133	−0.026
		CFC → SOP → ASMC→REBS	0.048	0.014	0.022	0.076
	Total effect	CFC → REBS	1.133	0.235	0.670	1.597

BoostSE, bootstrap standard error; LLCI, low limit for confidence interval; ULCI, upper limit for confidence interval.

### Network analysis

**Figure 5:**
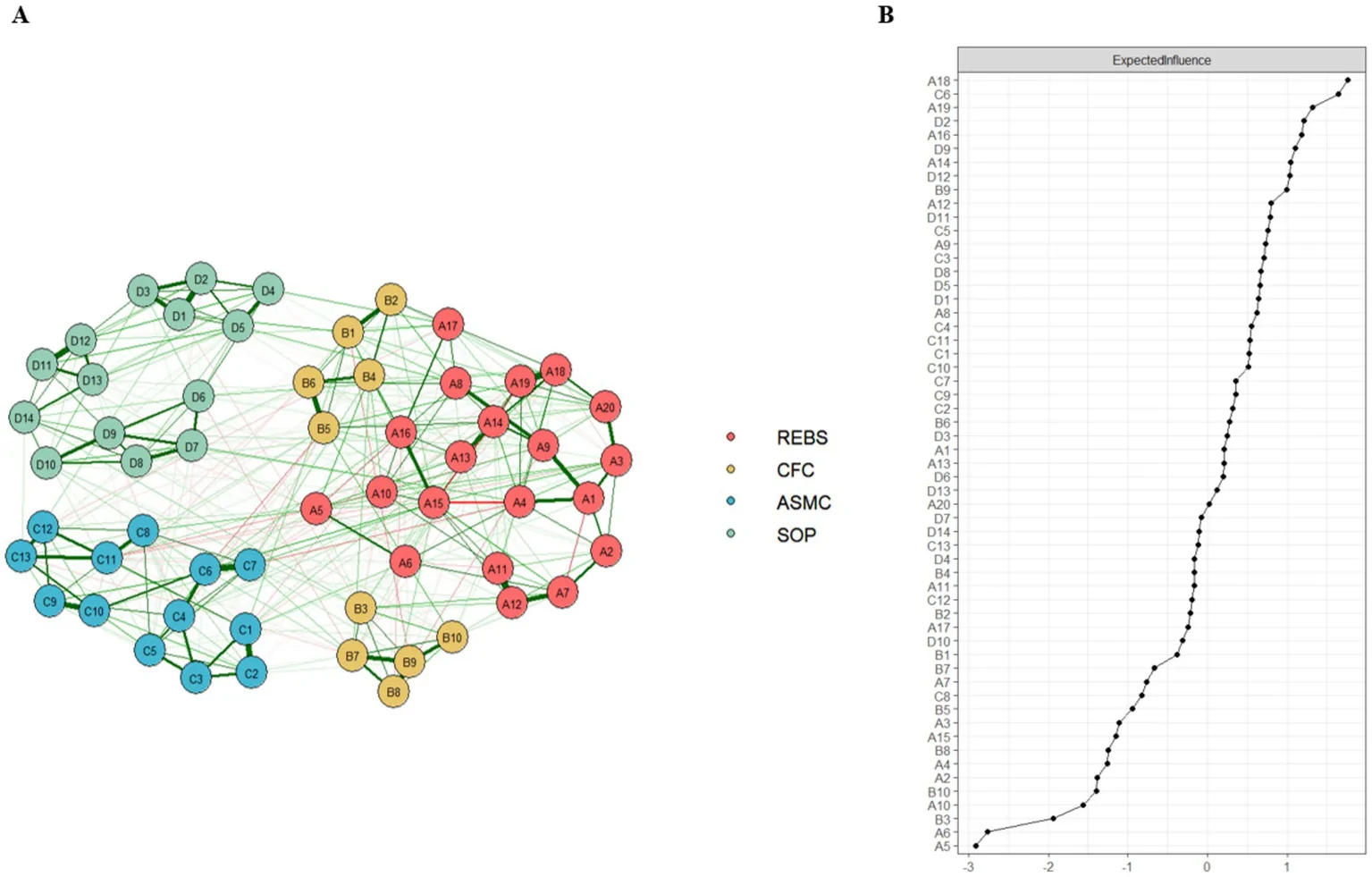
EBICglasso model based on the item-level network analysis **(A)** and network analysis centrality measure **(B)**.

The network comprised 57 nodes and 811 edges, representing 50.8% of all possible connections (Figure 5A). The correlation matrix is provided in Supplementary Figure S1. Network topology revealed distinct structural characteristics within REBS subnetwork: (1) REBS: The strongest association was observed between A18 and A19, followed by the link between A11 and A12; (2) CFC: The B8–B9 edge was the most prominent, succeeded by the B5–B6 connection; (3) ASMC: The C6–C7 pairing exhibited the highest centrality, with C1–C2 ranking second; and (4) SOP: The D11–D12 edge was the strongest, followed by the D1–D2 link. Abbreviations for all variables are detailed in Supplementary Table S6.

As shown in Figure 5B, node A18 exhibited the highest EI in the REBS subnetwork, whereas A5 showed the lowest. Within the CFC subnetwork, B9 demonstrated the highest EI, and B3 the lowest. In the ASMC subnetwork, C6 had the highest EI, and C8 the lowest. For the SOP subnetwork, D2 displayed the highest EI, and D10 the lowest. The results of the centrality difference test indicated that these nodes were statistically stronger than others. Specifically, A18 was the most influential node across the entire network, while A5 was the least influential.

**Figure 6:**
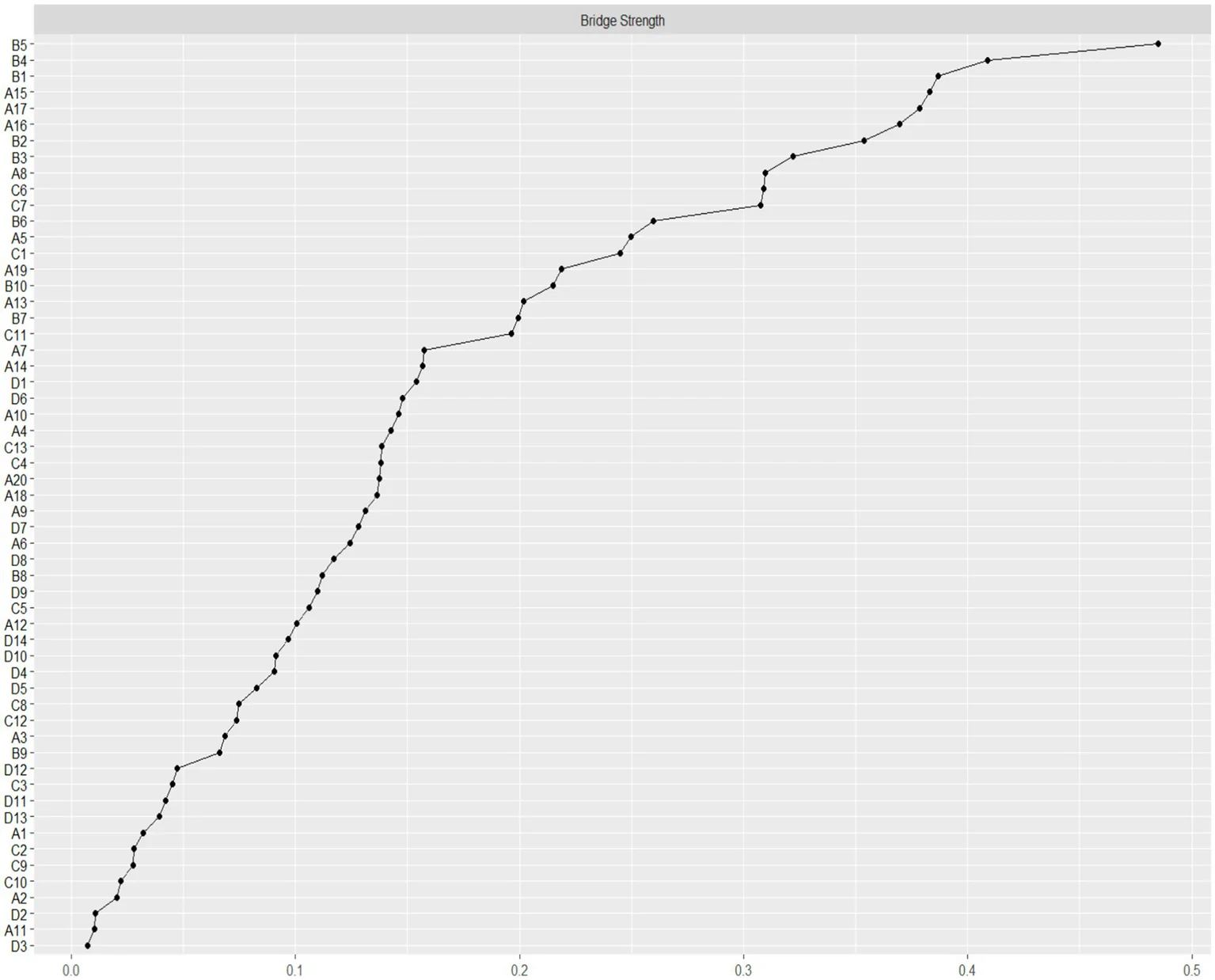
Bridge strength plot.

Figure 6 presents the bridge strength of the network. Nodes B5 and B4 exhibited the highest bridge strength, indicating that these nodes likely function as bridge nodes within the network. These nodes with high bridge centrality are the most influential in the network and are referred to as bridge symptoms. The results of bridge node centrality differences (Supplementary Figure S2) revealed significant distinctions between bridge nodes and peripheral nodes.

**Figure 7:**
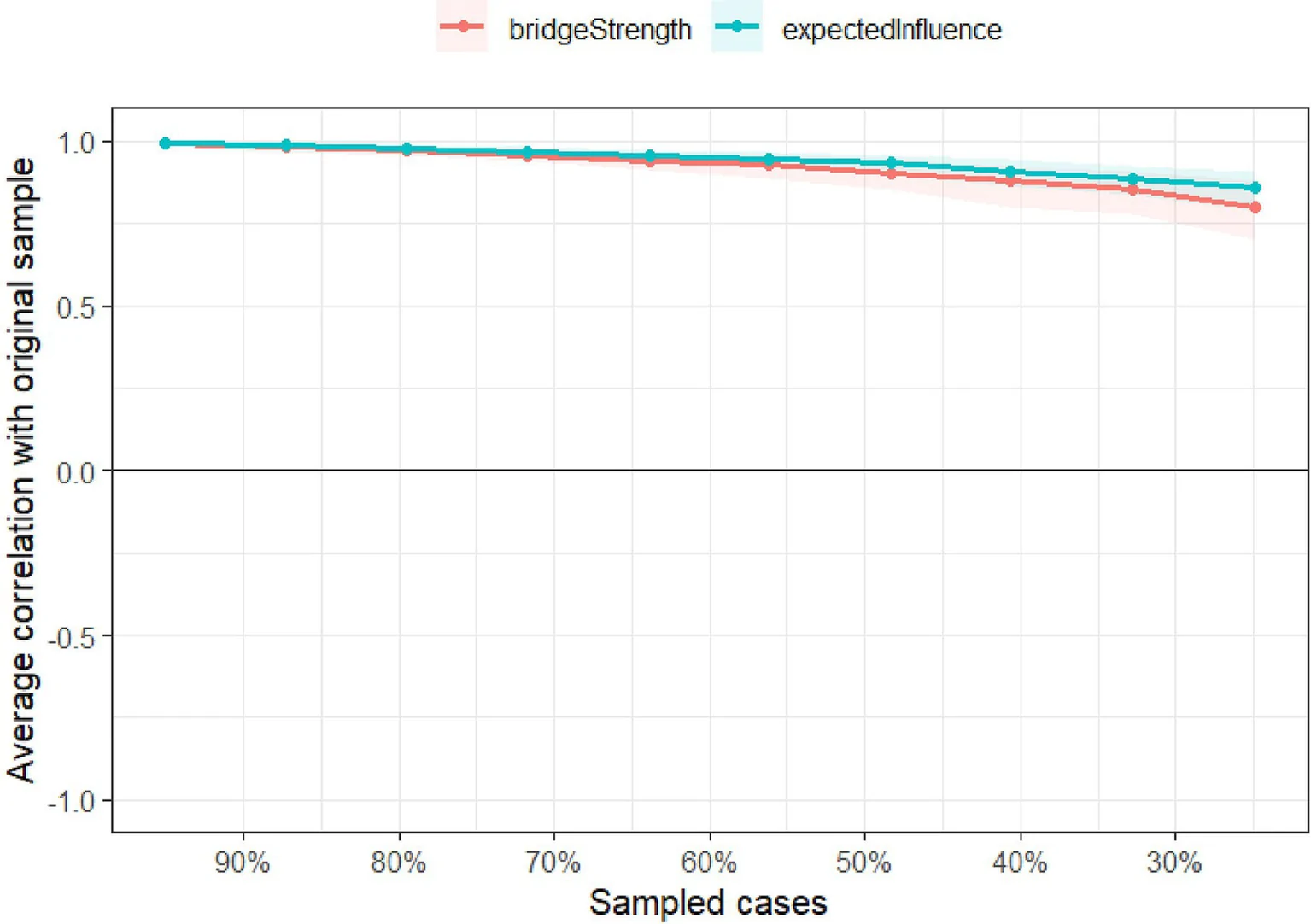
Stability of the expected influence and bridge strength of nodes.

Supplementary Figure S3 examines the accuracy of edge estimates via 95% bootstrapped confidence intervals. The relatively narrow intervals indicate good estimation accuracy and reliable network model results. Figure 7 presents the results of the case-dropping bootstrap test. The CS-C for EI centrality was 0.75. This indicates that the order of EI centrality between the original network and the networks re-estimated with reduced sample sizes was preserved (r ≥ 0.75) when up to 75% of the cases were excluded. Similarly, the CS coefficient for bridge strength was 0.75, suggesting that bridge strength estimates were sufficiently stable.

## Discussion

Eating motivation is influenced by a number of factors, such as self-esteem (Chammas et al., 2024), autonomous regulation of eating, and exercise (Kato et al., 2022). In this study, nearly half of college students have medium level of REBS. SOP and ASMC were associated with CFC and REBS. However, the association involving ASMC was not observed among students with normal weight. BMI was most prominent in the high REBS, reflecting weight differences. CFC also reflected significance from the low REBS, indicating future planning is concentrated among high REBS (Buchmann et al., 2023). ASMC showed the high REBS was significantly higher than others, while medium and low groups did not differ, suggesting appearance concern clusters in high REBS (Batool and Quratulain, 2023). In SOP, high REBS significantly outperformed the low group and all pairwise comparisons were significant, reflecting a continuous hierarchical distribution of goal orientation (Hsu et al., 2025). In addition, mediation analysis was conducted within a cross-sectional framework. Therefore, temporal precedence could not be established, and results should be interpreted as associational indications rather than causal effects.

In the present study, the majority of participants demonstrated a moderate level of REBS among college students. Meanwhile, a high level of REBS was more pronounced in females. Previous study has suggested that women who are more autonomous tend to demonstrate a sense of self, which has been associated with a reduced inclination to engage in unhealthy strategies (e.g., strict dietary restrictions; Pelletier et al., 2004a, 2004b). Furthermore, individuals with low level of REBS tended to have higher body weight in this study. A previous study has found that a lack of adherence to a healthy diet has been linked to a higher prevalence of overweight and obesity (Hur et al., 2021). Meanwhile, the association between CFC and REBS involving ASMC was not observed among students with normal weight. Evidence suggests that higher ASMC is associated with higher body surveillance and body shame among adolescents (Choukas-Bradley et al., 2020). The weight status of youth (i.e., overweight and obesity) is strongly related to body image. Specifically, research consistently shows that greater BMI is associated with heightened weight concerns among adolescents (Reel et al., 2015).

This study’s primary aim was to examine the CFC, SOP, and ASMC of college students within the framework of SDT. As expected, the chain-mediated model revealed a direct and positive association between CFC and REBS. This study found that CFC was positively associated with REBS, consistent with previous studies (Bénard et al., 2024; Liu, 2020). It is important to note that although several mediation effects reached statistical significance, their magnitudes were modest. This may be attributed to the fact that college students’ eating behaviors are not solely associated with individual motivation but also with external factors such as campus environments (Jurado-Gonzalez et al., 2025). Furthermore, self-reported CFC does not always translate into actual behavior. Consequently, while CFC is associated with healthy eating, it should not be viewed as a unique predictor (Dakanalis et al., 2023). Nevertheless, even modest effects may retain practical relevance at the population level. For instance, small differences in CFC or SOP may accumulate across individuals and coincide with variations in public health strategies concerning eating behaviors. These findings suggest that college students who reported clearer consideration of future outcomes also tended to report higher levels of REBS. SDT (LaCaille et al., 2020) also emphasizes the role of autonomous motivation in behavior sustainability. Individuals who engage in healthy eating behaviors with greater autonomous motivation and future orientation may be more likely to maintain such behaviors. The mediation analysis showed that SOP and ASMC partially explained the connection between CFC and REBS, which indicated that CFC was positively associated with REBS through positive SOP and high ASMC, supporting Hypothesis 1.

Our study showed that SOP was positively related to CFC. The CFC outcomes have been shown to be positively associated with SOP (Malin, 2022; Wigfield and Cambria, 2010). The effect size was modest, but this may reflect the complexity of real-world constraints (Sai Sushma et al., 2025). Persistent behaviors like high BMI reflect a misalignment between long-term health goals and current actions (Sai Sushma et al., 2025). CFC outcomes correspond to a tendency to prioritize long-term health (Hill and Pfund, 2025). Likewise, SOP aligns with increased confidence and motivation regarding healthy eating goals. Individuals are more likely to put in the effort necessary to achieve their goals when they set specific, measurable, and challenging goals. This has been shown to be associated with better quality of life and health (Keller et al., 2008). Consideration of future outcomes corresponds to a tendency to prioritize long-term health outcomes. Likewise, SOP aligns with increased confidence and motivation regarding healthy eating goals. People with a clear plan for the future and strong SOP reported greater adherence to specific healthy eating strategies, supporting Hypothesis 2.

Moreover, as expected, SOP was positively associated with ASMC, and this linkage was consistent with REBS. Previous research has demonstrated that future-oriented thinking is associated with stronger SOP (Joshanloo, 2024) and enhanced self-development (Kato et al., 2022). Individuals with SOP are more proficient at leveraging social media in ways that align with their personal goals. Conversely, those with a lower SOP are more prone to engaging in unproductive practices on social media (Twenge et al., 2018). These include perusing irrelevant content and seeking instant gratification through the medium, which may prove detrimental to their long-term objectives. Individuals with strong SOP tended to report greater proficiency in using social media in ways that corresponded with higher REBS, supporting Hypothesis 3.

Drawing on SDT, which proposes that individuals who hold positive future expectations often report higher autonomy, clearer goal setting, and greater self-direction, whereas excessive or maladaptive social media use has been associated with adverse health outcomes (Lee et al., 2022; Yang et al., 2022). This pattern aligns with the negative associations of controlled motivation in SDT (Deci and Ryan, 2000). Moreover, these findings correspond to previous research documenting significant associations between social media use and disordered eating (Barakat et al., 2023). Both eating disorder symptomatology and problematic social media engagement represent considerable challenges for young people (Mushtaq et al., 2023; Wilksch et al., 2019). The internalization of thin-ideal and appearance comparisons emerged as a relevant factor that may be related to appearance problems and eating disorders in all four countries (Kakar et al., 2023). While SOP may be associated with ASMC in the general population, the overall effect of CFC seems to operate through the alleviation of ASMC to bolster REBS. Conversely, excessive focus on high ASMC is likely linked to diminished REBS, potentially driven by body dissatisfaction, social comparison, or restrictive dieting pressures. In the normal weight population, although ASMC showed no significant association with REBS, its presence still coincided with a link between CFC and REBS. Conversely, in the overweight and obese population, ASMC was associated with REBS. Individuals exhibited a preoccupation with appearance that often overshadowed their pursuit of health. Consequently, REBS appeared unsupported and was inversely associated with excessive ASMC. Generally, this pattern is consistent with a suppression effect: although CFC was positively associated with REBS, it exhibited a negative association with ASMC, yielding a negative indirect effect. CFC lowers maladaptive ASMC, which in turn supports REBS. The internalization of this thin ideal was associated with a constellation of unhealthy eating habits and extreme weight-control behaviors, including excessive dieting, binge eating, and compulsive exercise, supporting Hypothesis 4.

This study focused on the associations of CFC, SOP, and ASMC with REBS within a mediation model framework, whereas the NA focused on the direct relationships among scale items (Borsboom, 2017, 2022; Robinaugh et al., 2020). Based on the interpretation of items, the study examined the structural relationships between CFC, SOP, ASMC, and REBS. In the NA, items A18, C6, and A19 showed high EI values, suggesting that these nodes occupy central positions within the network structure. In particular, B5 and B4 were not only central nodes but also served as key bridge nodes, exhibiting relatively bridge strength. This suggested that B5 and B4 represented important connections among CFC, SOP, ASMC, and REBS. From the perspective of SDT, these nodes may represent the integration of autonomous and controlled motivational processes. Specifically, B4 and B5 reflect the cognitive elaboration of long-term health outcomes, which aligns with SDT’s conceptualization of autonomous regulation. It suggests that individuals engage in health behaviors out of personally endorsed values rather than external pressure. This represents an important step toward autonomous motivation, as it involves the internalization of extrinsic values into one’s self-concept. Although this process has been theorized in previous SDT health behavior literature, it is rarely operationalized at the cognitive level. SDT posits that autonomous regulatory processes are more predictive of sustained health behavior than controlled forms of motivation, but the effect sizes are often modest and variable across behaviors and populations. Evidence indicates that autonomous motivation is associated with more effective and sustained health behavior change. Notably, its effect size is strongest in physical activity domains compared to educational, occupational, or clinical contexts, and diminishes with age, yielding weaker effects in older populations (Hagger, 2025; Ntoumanis and Moller, 2025; Slemp et al., 2024). A18 and A19 reflected the perception of long-term health benefits, aligning with an autonomous regulatory mechanism within SDT. They capture the importance of health behavior outcomes, representing the affective-cognitive integration of wellness values that characterize integrated regulation. And C6 captured the salience individuals assign to their external image in the digital age (Dinh and Lee, 2025). It specifically captures the co-occurrence of contingent self-worth and externally referenced standards within the social media context. This node occupies a central position linking physical appearance concerns and social recognition, characterizing health behaviors as cognitively entangled with social approval rather than solely reflecting authentic self-expression (Dortyol et al., 2026). From the perspective of SDT, it is evident that individuals demonstrate divergent motivational trajectories depending on whether the drivers are autonomous or controlled. So when centered on social interaction or entertainment, individuals are more likely to engage in self-presentation (Gao et al., 2022). These patterns coincided with higher scores on future-oriented goals, reflecting aspirations not only for health but also for future wellbeing and current social recognition (Yang et al., 2025). This pattern is consistent with the view that long-term goals are linked to higher intrinsic motivation for health-related behaviors. According to SDT, people have a fundamental need to form authentic connections with others (Mushtaq et al., 2023; Wilksch et al., 2019). When individuals receive “likes” or comments, their needs for belonging and recognition are addressed, which has been associated with more internalized forms of motivation for healthy eating.

This study offers several strengths. First, it documents the sequential associations linking CFC and REBS via SOP and ASMC, findings which may inform the development of targeted interventions to support improvements in dietary behaviors among students. Second, by examining both SOP and ASMC, this work extends SDT to encompass a broader range of motivational dynamics in dietary regulation. Furthermore, the use of NA provides a nuanced mapping of the interplay among REBS, CFC, ASMC, and SOP.

This research also had certain shortcomings. First, the cross-sectional nature of this study precludes temporal precedence, restricting our interpretations to contemporaneous associations rather than causal effects. Second, the measurement model demonstrated suboptimal fit indices, suggesting potential misspecification and limiting the precision of parameter estimates. Third, our study used only the two scales associated with the CFC and the REBS. It may not capture its full complexity, including the differences between them. Fourth, we had an overrepresentation of female participants (81.1%) in this study and recommend that future studies should aim to recruit gender-balanced samples, as this study limits the generalizability of the findings to male college students and broader populations. Fifth, selection bias arising from convenience sampling and voluntary online recruitment limits generalizability; the sample may not fully represent all college students in Wuhan, particularly underrepresenting those with lower internet accessibility or willingness to participate. Additionally, Reliance on self-reported questionnaires may have led participants to underreport undesirable behaviors and misremember past habits, potentially compromising the findings.

Future studies should employ multi-method assessments to enhance measurement results. Integrating diverse motivational scales alongside behavioral data and qualitative interviews would provide a more comprehensive understanding of the complex associations underlying food choice. Furthermore, given that the link between CFC and REBS is likely reciprocal, subsequent studies should adopt longitudinal or cross-lagged designs. This would allow for the examination of bidirectional pathways, addressing the current limitation of focusing solely on unidirectional associations.

## Conclusion

Based on the NA, interventions should adopt a multi-pathway strategy, using the bridge nodes (B5 and B4) as levers to influence the central nodes (A18, A19 and C6) via the identified associative pathways. First, interventions should aim to foster future-oriented thinking in daily food choices. Second, these initiatives should align such prospective cognition with personal goals, emphasizing the association between healthy eating and subjective wellbeing. Finally, challenge-based activities can guide individuals to shift social media engagement from passive comparison toward curating a health-positive identity. In summary, a multi-pathway approach leverages key nodes to facilitate cognitive and behavioral shifts by integrating future-oriented framing with identity construction.

## Data Availability

The original contributions presented in the study are included in the article/Supplementary material, further inquiries can be directed to the corresponding author.
