# Supplementary material for: The interaction between future food considerations, regulation of eating behaviors, sense of purpose, and social media consciousness among college students: a study on mediation effects and network analysis
Source: Front Psychol. 2026 Aug 12;17:1827219. doi: 10.3389/fpsyg.2026.1827219 (PMC13506398; doi:10.3389/fpsyg.2026.1827219)
Supplement: Supplementary file 1 [file Supplementary_file_1.docx]

SUPPLEMENTARY DATA

The Interaction between Future Food Considerations, Regulation of Eating Behaviors, Sense of Purpose, and Social Media Consciousness among College Students: A Study on Mediation Effects and Network Analysis

Table S1 Multiple comparisons of demographic variables

| Variables | Group (I) | Group (J) | Mean difference (I-J) | Post-hoc (Letters) | Levene’s Test | Welch’s ANOVA |
| --- | --- | --- | --- | --- | --- | --- |
| Age | - | - | - | - | 3. 211* | 2. 578 |
| BMI | High | Medium | -0. 039 | a | 11. 010*** | 3. 918* |
|  |  | Low | -2. 719* | a |  |  |
|  | Medium | Low | -2. 680* | b |  |  |
| CFC | High | Medium | 0. 762 | a | 6. 936** | 29. 890*** |
|  |  | Low | 3. 481*** | b |  |  |
|  | Medium | Low | 2. 719*** | b |  |  |
| ASMC | High | Medium | 8. 997*** | a | 8. 931*** | 33. 001*** |
|  |  | Low | 10. 540*** | a |  |  |
|  | Medium | Low | 1. 543 | b |  |  |
| SOP | High | Medium | 5. 697*** | a | 6. 857** | 67. 069*** |
|  |  | Low | 14. 287*** | b |  |  |
|  | Medium | Low | 8. 589*** | c |  |  |

Note: Groups sharing the same superscript letter (a, b, c) are not significantly different at P< 0.05, and groups with different letters differ significantly. *P< 0.05; **P< 0.01; ***P< 0.001.

Table S2 The correlation analysis of the influencing factors (N=778)

|  | Mean ± SD | REBS | CFC | ASMC | SOP |
| --- | --- | --- | --- | --- | --- |
| REBS | 100. 37±14. 55 | 1 |  |  |  |
| CFC | 31. 17±4. 58 | 0. 330** | 1 |  |  |
| ASMC | 53. 78±14. 80 | 0. 340** | -0. 036 | 1 |  |
| SOP | 68. 44±11. 78 | 0. 527** | 0. 255** | 0. 269** | 1 |

Note: **P* <0. 05; ***P* <0. 01; ****P* <0. 001.

Table S3 Multicollinearity diagnosis of covariates

| Variables | VIF | 1/VIF |
| --- | --- | --- |
| Age | 1. 001 | 0. 999 |
| BMI | 1. 001 | 0. 999 |

Table S4 The relationship of REBS, CFC, ASMC and SOP (N=778)

|  | Model l ^a^ | | Model 2 ^b^ | |
| --- | --- | --- | --- | --- |
| Variables | β | 95%CI | β | 95%CI |
| CFC | 0. 237 | (0. 567~0. 938)*** | 0. 238 | (0. 570~0. 942)*** |
| ASMC | 0. 240 | (0. 178~0. 293)*** | 0. 240 | (0. 179~0. 294)*** |
| SOP | 0. 402 | (0. 422~0. 571)*** | 0. 403 | (0. 423~0. 573)*** |

a: crude model

b: adjusted gender and BMI

Note:**P*＜0.05; ***P* <0. 01; ****P* <0. 001.

Table S5 Structural Equation Model Fit Index

|  | χ^2^/df | CFI | TLI | RMSEA | SRMR |
| --- | --- | --- | --- | --- | --- |
| Reference | <3 | > 0. 95 | > 0. 95 | <0. 06 | <0. 08 |
| Model 1 | 4. 43 | 0. 718 | 0. 707 | 0. 066 | 0. 084 |
| Model 2 | 2. 99 | 0. 710 | 0. 698 | 0. 069 | 0. 088 |
| Model 3 | 2. 70 | 0. 615 | 0. 599 | 0. 092 | 0. 102 |

Note: Model 1: total (n=778); Model 2: normal weight (n=422); Model 3, overweight and obesity (n=202); CFI, Comparative Fit Index; TLI, Tucker-Lewis index; RMSEA, Root Mean Square Error Of Approximation; SRMR, Standardized Root Mean Square Residual.

Figure S1 Bootstrapped diﬀerence test for EI in item-level network

Table S6 The abbreviation for each variable selected in the item-level network

| Variables |  |
| --- | --- |
| REBS | A1: Preparing healthy food makes me happy. |
|  | A2: Healthy eating is what others expect of me. |
|  | A3: I enjoy trying new cooking methods to prepare healthy food. |
|  | A4: Healthy eating is a way to ensure a healthy life in the long term. |
|  | A5: I feel that I must be thin. |
|  | A6: I don't want to feel inferior about my appearance or body shape. |
|  | A7: Other people require me to eat healthily. |
|  | A8: Healthy eating is part of my lifestyle. |
|  | A9: Healthy eating makes me feel satisfied. |
|  | A10: Unhealthy eating makes me feel guilty. |
|  | A11: If I don't eat healthily, the people around me (e.g., partner or parents) will be unhappy. |
|  | A12: People around me nag me to eat healthily. |
|  | A13: I think it is worthwhile to try to control my eating behavior. |
|  | A14: Healthy eating is one of the lifestyles I want. |
|  | A15: If I cannot control my eating behavior, others will laugh at me. |
|  | A16: Controlling my eating behavior has become my habit. |
|  | A17: I believe my healthy eating is consistent with other healthy behaviors (such as exercise). |
|  | A18: I believe healthy eating will ultimately make me feel better. |
|  | A19: I believe healthy eating is something that makes me feel better. |
|  | A20: I think cooking healthy food is fun for me. |
| CFC | B1: I consider my future health status and try to influence it through my daily eating behaviors. |
|  | B2: To achieve my health goals many years from now, I often adopt specific eating behaviors. |
|  | B3: I choose food only to meet my current needs; as for the future, I let it take its course. |
|  | B4: For the sake of future health, I am willing to sacrifice the immediate pleasure brought by current eating. |
|  | B5: I believe we should always be vigilant about the consequences of poor eating behaviors, even if these consequences may not appear until many years later. |
|  | B6: I believe adopting eating behaviors beneficial to long-term health is more important than satisfying the current craving for delicious food. |
|  | B7: I rarely consider the potential threat of eating behaviors to my future health. |
|  | B8: I don't think it's necessary to avoid eating certain foods now; I will deal with future matters later. |
|  | B9: I choose food only to satisfy my current needs; I will deal with future matters later. |
|  | B10: For me, the immediate consequences of daily eating are more important than its long-term consequences. |
| ASMC | C1: When others take photos of me, I think about how I will look in the photos if they are posted on social media. |
|  | C2: When others browse my photos on social media, I think about how specific parts of my body look. |
|  | C3: Even when I am alone, I imagine how I would look in social media photos. |
|  | C4: During the day, I spend time thinking about how attractive I am when others browse my photos on social media. |
|  | C5: I try to guess how people on social media will react to my appearance in photos. |
|  | C6: Whether I am attractive in photos is more important than other things I do on social media. |
|  | C7: When attending social events, I care more about whether I am attractive in photos that others post on social media than whether I am having fun. |
|  | C8: If an unflattering photo of me is posted on social media, I feel uncomfortable. |
|  | C9: I repeatedly look at my own photos on social media. |
|  | C10: I zoom in on photos on social media to see how specific parts of my body look. |
|  | C11: If someone takes a photo of me that might be posted on social media, I will ask to see it first to ensure I look good. |
|  | C12: Before posting photos on social media, I edit them or add filters to make myself look better. |
|  | C13: If someone takes a photo of me that might be posted on social media, I intentionally strike a flattering pose to make myself look more attractive. |
| SOP | D1: My life purpose is clear. |
|  | D2: I have determined my life purpose. |
|  | D3: I can describe my life purpose. |
|  | D4: I am confident about my life purpose. |
|  | D5: I have sufficient understanding of my life purpose. |
|  | D6: I hope to make a contribution to the organization I belong to. |
|  | D7: I desire to help others. |
|  | D8: I serve society in many ways, both large and small. |
|  | D9: I want to use my life to make a positive impact on society. |
|  | D10: I strive to promote the well-being of others. |
|  | D11: The ultimate purpose of my life is gradually becoming clearer. |
|  | D12: I am becoming increasingly clear about my life purpose. |
|  | D13: I am discovering the deeper purpose of my life. |
|  | D14: What I have been doing recently is very meaningful for finding my life purpose. |

Figure S2 Bootstrapped difference test for bridge strength in item-level network

Figure S3 Accuracy of edge weights in item-level network
